# Emergence of the invasive malaria vector *Anopheles stephensi* in Khartoum State, Central Sudan

**DOI:** 10.1186/s13071-021-05026-4

**Published:** 2021-10-02

**Authors:** Ayman Ahmed, Rua Khogali, Mohammed-Ahmed B. Elnour, Ryo Nakao, Bashir Salim

**Affiliations:** 1 The World Health Organization, 1211 Geneva, Switzerland; 2 Department of Parasitology, Faculty of Veterinary Medicine, University of Khartoum, P.O. Box 32, Khartoum North, Sudan; 3 Department of Parasitology & Medical Entomology, Tropical Medicine Research Institute, National Center for Research, P.O. Box 1304, 11111 Khartoum, Sudan; 4 Laboratory of Parasitology, Faculty of Veterinary Medicine, Graduate School of Infectious Diseases, Hokkaido University, Sapporo, Japan

**Keywords:** *Anopheles stephensi*, Emergence, Invasive diseases vector, Asian malaria vector, Malaria, Sudan

## Abstract

The emergence of the Asian invasive malaria vector, *Anopheles stephensi*, has been identified in Khartoum, the capital city of Sudan. This is the first report that confirms the geographical expansion of this urban mosquito into Central Sudan. We urgently recommend the launch of a national entomological survey to determine the distribution of this invasive disease vector and to generate essential information about its bionomics and susceptibility to available malaria control measures.

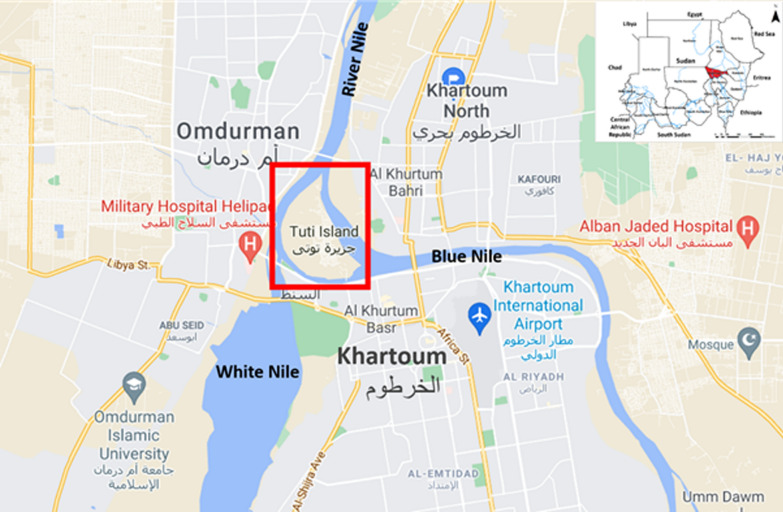

*Anopheles stephensi* is an invasive disease vector that is originally endemic in Asia and competent in transmitting both malaria parasites *Plasmodium falciparum* and *P. vivax* [1]. *Anopheles stephensi* has recently invaded Africa, specifically the Horn of Africa [2]. It was first reported in Djibouti (2012) and Ethiopia (2016) [3, 4]. Furthermore, in 2019, this mosquito species was detected in the coastal and sub-coastal regions of the Red Sea in Sudan [5]. Considering the heavy burden and high risk of malaria in Africa, with more than 94% of the 229 million globally estimated cases in 2019 being reported from Africa, the majority of cases were in individuals living in the sub-Saharan region, which includes Sudan [6]. The establishment of this competent malaria vector in Africa is of global importance. The World Health Organization (WHO) has raised an alarm about the invasion and spread of *An. stephensi* into Africa to urge national malaria control programmes and their partners in areas at risk to be vigilant and to improve and upscale their surveillance systems for the early detection and control of this invasive mosquito species [5].

During a fieldwork assignment that focused on collecting *Culex* and *Aedes* mosquitoes from Tuti Island (15.6202° N, 32.5062° E; Khartoum, capital city of Sudan) between August and September 2018 (Fig. 1), 21 unknown *Anopheles* mosquitoes were aspirated. These were identified to species level using standard morphological keys [7].

**Fig. 1:**
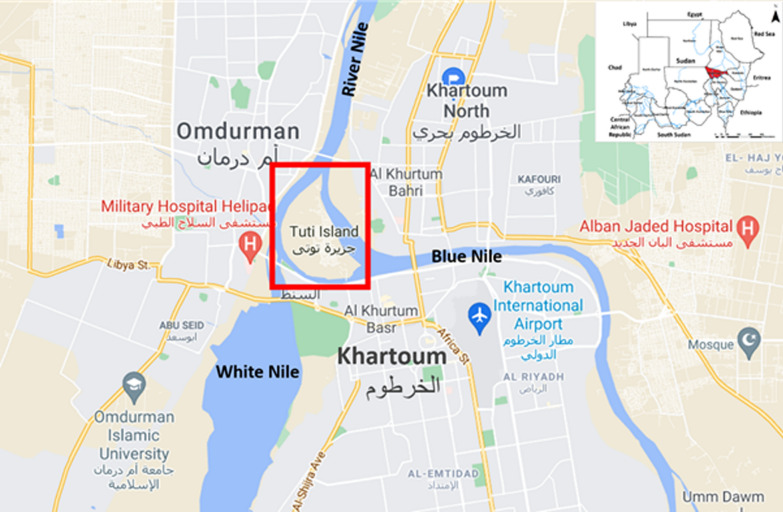
Inset map shows the location of the *Anopheles* mosquito samples collected (red square)

Of the mosquitoes collected from Tuti Island, the majority (19; 91%) of the samples were identified as *An. arabiensis*, the major malaria mosquito vector in Sudan [8]. However, the remaining two samples (9%) were morphologically identified as *An. stephensi*. Considering that *An. stephensi* has never been reported before in Sudan except for the Red Sea and Gedaref states in 2019, further confirmatory steps were essential. We extracted the total DNA from all 21 *Anopheles* mosquito samples using DNAzol (Molecular Research Center, Inc., Cincinnati, OH, USA) according to the manufacturer’s guidelines. Our genetic analysis confirmed the morphological identification of the mosquitoes by sequencing the cytochrome c oxidase I (*COI*) gene [9, 10]. Phylogenetic analysis suggests that *An. stephensi* from Sudan is closely related to the *An. stephensi* from Ethiopia (Fig. 2).

**Fig. 2:**
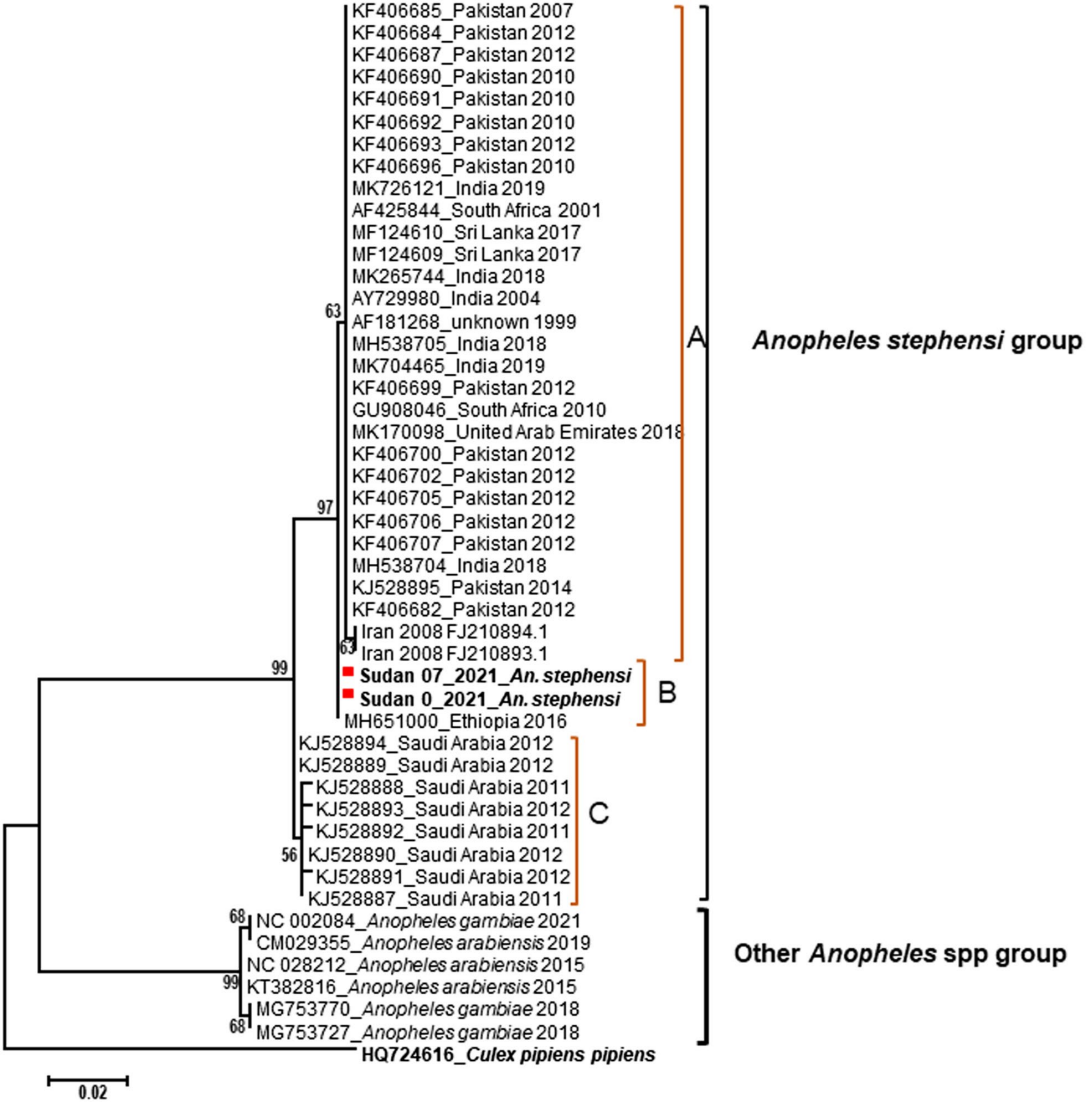
Phylogenetic tree of the maximum likelihood generated using *COI* sequences from Khartoum (marked with the red box) and other countries from which data are available

This is the first report of *An. stephensi* in Khartoum State, Central Sudan. Our serendipitous finding suggests this mosquito species has spread beyond the coastal regions and into Sudan’s interior, thus highlighting the pressing need to conduct a countrywide survey. Such a survey would help establish an accurate distribution map for this disease vector and would provide data to predict further species invasion into the five neighbouring *An. stephensi*-free countries: the Central African Republic, Chad, Egypt, Libya, and South Sudan. Recently developed prediction models based on current *An. stephensi* distribution patterns and habitat suitability have indicated high potential risk for the spread of *An. stephensi* unless very strict control measures are rapidly adopted alongside targeted entomological surveillance [2]. Previous studies warned of the serious threat of unprecedented epidemics of malaria in large cities in Africa, including Khartoum, if they were invaded by *An. stephensi* [11], particularly because the national vector surveillance systems in Africa are experienced and oriented toward the rural endemic vectors, not the urban *An. stephensi* [11].

The invasion of *An. stephensi* populations into the Republic of Djibouti in 2012 was associated with several urban malaria epidemics. The disease was controlled by combination of larvicides, thermal fogging of insecticides, and habitat sanitation [3, 12]; other studies warn that similar scenarios might develop throughout Africa if this vector continues to spread into other densely populated urban areas [11]. It is unknown yet whether the recent (2018 and 2019) malaria epidemic in South Kordofan, Sudan, is linked to the establishment of a new *An. stephensi* population in the area [13]. The serious threat of malaria epidemics in Africa due to the spread of *An. stephensi* in the area prompted WHO to issue a vector alert in 2019 [5]. This alert was raised to encourage the African countries that already had established populations of *An. stephensi*, or that shared borders with countries reporting the presence of this vector, to update their strategies and guidelines for mosquito surveillance and control [5]. Furthermore, WHO aimed to mobilize local and international resources to improve their national vector surveillance system and to increase the capacity of their surveillance and control teams in order to implement surveys for the early detection and reporting of *An. stephensi*. It is important to gather data and fill current information gaps on *An. stephensi* bionomics in Africa, including the feeding and resting behaviours and preferences. Determining how susceptible these populations are to the locally implemented vector control measures is also important in order to deliver a timely and effective response that averts the local establishment and further spread of *An. stephensi* [5]. Considering the zoophilic nature of *An. stephensi*, it might be useful to adopt innovative and environmentally friendly vector control tools such as endectocides including nitisinone and ivermectin for the control of this species [14, 15]. Strict implementation of the International Health Regulations (IHR 2005) is essential to prevent this mosquito from invading other *An. stephensi*-free countries [5].

Further studies are needed to investigate the risk factors in Sudan that influence the spread of *An. stephensi* and also to target the possible invasion routes to improve disease prevention and control interventions [16]. Surveillance systems in the countries with confirmed presence and/or high risk of *An. stephensi* emergence could use satellites to produce evidence-based habitat suitability prediction models for early detection [2]. More importantly, molecular and genetics-based tools should be incorporated into the national vector surveillance systems to establish an early warning/response system that would quickly detect the introduction of invasive disease vectors before they adapt and establish locally [3, 4, 17].

In conclusion, in this report we document the first detection of the invasive Asian malaria-transmitting vector *An. stephensi* into Khartoum State, Central Sudan. The arrival of this mosquito is of high public health concern due to the threat of urban malaria outbreaks in the densely populated state of Khartoum. We strongly emphasize the need to deploy a national vector survey that targets *An. stephensi* in order to (1) determine the geographical distribution of this disease vector across the country, (2) provide evidence on feeding and resting behaviours in relation to its susceptibility to current vector control measures, and (3) identify how *An. stephensi* contributes to local malaria transmission. Such a survey should be informed by habitat suitability prediction models and supported with genomic tools. We urge the Sudan Ministry of Health, malaria stakeholders, and their partners to mobilize resources and implement a strategic prevention and control action plan to prevent the local establishment and/or further spread of this invasive vector in the country and the region. Several measures need to be in place for the success of such an action plan, including upscaling the surveillance system and implementing intensive surveys, deploying effective vector control interventions, and strictly implementing the International Health Regulations.

## Data Availability

The datasets supporting the conclusions of this article are included within the article. Our *An. stephensi* DNA sequences were submitted to GenBank under accession numbers (Submitted to GenBank waiting for the accession numbers).
