# Correction to: Emergence of the invasive malaria vector *Anopheles stephensi* in Khartoum State, Central Sudan

**DOI:** 10.1186/s13071-021-05074-w

**Published:** 2021-11-01

**Authors:** Ayman Ahmed, Rua Khogali, Mohammed-Ahmed B. Elnour, Ryo Nakao, Bashir Salim

**Affiliations:** 1 Institute of Endemic Diseases, University of Khartoum, Khartoum, 11111 Sudan; 2 Department of Parasitology, Faculty of Veterinary Medicine, University of Khartoum, P.O. Box 32, Khartoum North, Sudan; 3 Department of Parasitology & Medical Entomology, Tropical Medicine Research Institute, National Center for Research, P.O. Box 1304, 11111 Khartoum, Sudan; 4 Laboratory of Parasitology, Faculty of Veterinary Medicine, Graduate School of Infectious Diseases, Hokkaido University, Sapporo, Japan

## Correction to: Parasites Vectors (2021) 14:511 10.1186/s13071-021-05026-4

Following publication of this article [1] it has come to our attention that the first author's affiliation is stated incorrectly. The correct affiliation for Ayman Ahmed is: Institute of Endemic Diseases, University of Khartoum, Khartoum 11111, Sudan. This has been corrected in the original article.

Concerning data availability, the authors wanted to take this opportunity to share the GenBank accession numbers of the *Anopheles stephensi* *COI* sequences generated in their study: MZ725449 and MZ725450.

Thank you for reading this correction.
